# Cognitive Pragmatic Rehabilitation Program in Schizophrenia: A Single Case fMRI Study

**DOI:** 10.1155/2017/1612078

**Published:** 2017-01-23

**Authors:** Ilaria Gabbatore, Francesca M. Bosco, Elisabetta Geda, Luigi Gastaldo, Sergio Duca, Tommaso Costa, Bruno G. Bara, Katiuscia Sacco

**Affiliations:** ^1^Faculty of Humanities, Research Unit of Logopedics, Child Language Research Center, University of Oulu, Oulu, Finland; ^2^Center for Cognitive Science (CSC), University and Polytechnic of Turin, Turin, Italy; ^3^Department of Psychology, University of Turin, Turin, Italy; ^4^Neuroscience Institute of Turin, Turin, Italy; ^5^Brain Imaging Group (BIG), Koelliker Hospital, Turin, Italy; ^6^Psychiatric Service, Mental Health Department, ASL-TO3, Turin, Italy; ^7^CCS fMRI, Koelliker Hospital, Turin, Italy

## Abstract

*Introduction.* The present study was intended to evaluate the effects of a rehabilitative training, the Cognitive Pragmatic Treatment (CPT), aimed at improving communicative-pragmatic abilities and the related cognitive components, on the cerebral modifications of a single case patient diagnosed with schizophrenia.* Methods.* The patient underwent two functional magnetic resonance imaging (fMRI) sessions, before and after the treatment. In order to assess brain changes, we calculated the Amplitude of Low Frequency Fluctuation (ALFF) index of the resting-state fMRI signal, which is interpreted as reflecting the intensity of the spontaneous regional activity of the brain. Behavioural measures of the patient's communicative performance were also gathered before and after training and at follow-up.* Results.* The patient improved his communicative performance in almost all tests. Posttraining stronger ALFF signal emerged in the superior, inferior, and medial frontal gyri, as well as the superior temporal gyri.* Conclusions.* Even if based on a single case study, these preliminary results show functional changes at the cerebral level that seem to support the patient's behavioural improvements.

## 1. Introduction

Communicative-pragmatic ability is a typically impaired feature in schizophrenia [1]. Communicative-pragmatic refers to the ability to use language and nonverbal expressive means, such as gestures and facial expressions, and to convey meaning in a given context [2]. Despite their almost intact syntactic ability [3], patients with schizophrenia show difficulties in a more complex use of language, that is, pragmatic [4]. They show poor performance in comprehending different kinds of pragmatic phenomena, such as indirect speech acts [5], deceit [6], irony [7], and metaphoric expression [8], and in recognizing and recovering communicative failures [9]. Moreover, schizophrenia implies deficits in the management of conversation [10], narrative impairments [11], and poor performance in nonverbal expressiveness [12] and in emotional prosody (see [13]). A broad assessment provided using the Assessment Battery for Communication (ABaCo) [14–16] has recently given an articulated description of communicative-pragmatic deficit in patients with schizophrenia [17]. Results of the study showed that patients have difficulties in using linguistic, extralinguistic, and paralinguistic expressive modalities both in the comprehension and in the production of various types of pragmatic tasks, as direct and indirect speech acts, irony and deceit. According to some authors (e.g., [18]) these deficits are related to the well-demonstrated theory of mind (ToM; [19]) impairment of these patients (e.g., [20, 21]). However, there is evidence in the literature to suggest that ToM is not able, alone, to fully explain the communicative deficits of these patients [9].

Pragmatic ability is something which is articulated and requires a broad interplay of processes that seem to involve several prefrontal, frontal, and temporal networks, even though the identification of specific neural substrates underlying this competence is quite complicated. Shibata et al. [22] investigated the neural substrates of irony comprehension, showing higher activation in the right medial prefrontal cortex and right precentral and left superior temporal sulcus. More recently, Akimoto et al. [23] highlighted the complexity of the neural correlates involved in the comprehension of irony. The authors identified the right anterior superior temporal gyrus as being associated with the representation of social conceptual knowledge, while the medial prefrontal cortex and the right anterior inferior temporal gyrus could be involved in the understanding of context. Uchiyama et al. [24], in an fMRI study, investigated the ability to distinguish between literal and intended meaning in understanding metaphor and sarcasm. The authors found metaphor-specific activation in the head of the caudate and sarcasm-specific activation in the left amygdala. Furthermore, both metaphor and sarcasm activated the anterior rostral medial frontal cortex. The authors concluded that these areas are jointly involved in monitoring the coherence of literal meaning of the utterance and in mentalizing within social contexts in order to understand the pragmatic-communicative meanings of an utterance.

Data in the literature show that cognitive and communicative impairments may restrict the possibilities for functional recovery and can persist also after the psychosis subsides (e.g., [25, 26]); moreover, individuals with schizophrenia seem to be able to improve their cognitive and social cognitive abilities following behavioural training [27]. Despite this evidence, little attention has been given to cognitive remediation in schizophrenia. Some authors have developed remediation programs to improve social functioning [28] and to affect recognition [29], awareness, motivation and social competencies [30], metacognition [31], and theory of mind [32].

We recently designed the Cognitive Pragmatic Treatment (CPT), an integrated treatment specifically focused on the remediation of communicative-pragmatic abilities and related cognitive components. It offers the participants the opportunity to train their own ability to manage communicative interchanges using different communicative means, that is, language and gestures and appropriate paralinguistic cues, and adhering to the social and conversational settings where the communicative interaction takes place. Additionally, the CPT focuses on components such as awareness, theory of mind, and executive functions, which play a role [11, 33] in structuring efficient communication. The training has already been used with TBI individuals [34, 35] and in a pilot study with patients with schizophrenia [36], revealing high rates of effectiveness in improving their communicative performance.

A training program is aimed at restructuring cerebral functioning. To measure brain changes in response to practice, functional magnetic resonance imaging (fMRI) has proven to be an excellent tool. It has been used to explore the cerebral mechanisms underlying specific training programs, with both healthy and clinical individuals in several domains. In as much as what concerns schizophrenic individuals, a recent review [37] summarized the studies which evidenced brain plasticity through neuroimaging. More specifically, what kind of changes could be expected in these patients after training of their communicative abilities? In recent years, alterations in neural oscillations have been identified as a potential mechanism involved in the pathophysiology of schizophrenia, able to explain cognitive dysfunctions and certain symptoms of this illness (for a review, see [38]). In particular, alterations in the Amplitude of Low Frequency Fluctuation (ALFF) have been found in adult patients with schizophrenia [39], in adolescents with early onset [40, 41], and, to a lesser extent, in adolescents and young people at high clinical psychotic risk [41]. Besides, these alterations covary with cognitive deficits [42], suggesting that the ALFF relates to symptoms and can predict an onset of schizophrenia. In a rehabilitation perspective, changes of the ALFF index can then give an indication of the cerebral mechanisms accompanying recovery. On the basis of these, we tested the effectiveness of the CPT on a single chronic patient with a diagnosis of schizophrenia. In particular we assessed the ALFF index before and after the CPT: the aim was to investigate the existing relationship between the behavioural improvements due to the training and the cerebral changes in functional activity. We hypothesized a posttreatment reorganization of regions involved in communication: this would indicate that CPT can favor brain plasticity towards improvement. To the best of our knowledge, there are no studies assessing the effectiveness of a specific training for the communicative-pragmatic abilities in patients with schizophrenia, using a functional brain activity measure.

## 2. Material and Methods

### 2.1. Profile of the Patient

Patient S.T., a 39-year-old male, right handed, with a high-school diploma as electronics technician (13 years of education), has been recruited from the Department of Mental Health (DSM-To2) in Turin. He had a diagnosis of paranoid schizophrenia, made by a qualified clinician belonging to the clinical unit, using DSM-IV criteria. S.T. had lived for a few years in a therapeutic center, that is, a health care structure where people with several kinds of intellectual and psychiatric disorders live with the constant help and assistance of qualified staff such as nurses, professional educators, and psychologists. Nevertheless, at the moment of the study he used to live in sheltered accommodation in Turin, a housing arrangement where a small group of people with similar therapeutic conditions live together receiving assistance from specialized health workers for just a few hours per week; moreover, he was included in a supported employment program, under the supervision of the local mental health care unit. The symptomatology of the patient at the time of the first assessment was investigated with the Positive and Negative Syndrome Scale (PANSS; [43]). The PANSS was completed by a qualified psychiatrist and reported 15 scores at the negative symptoms scale, 15 scores at the positive symptoms scale, and 36 scores at the general symptoms scale (PANSS total score: 66). S.T. was treated with atypical antipsychotics (risperidone), anxiolytics (chlordesmethyldiazepam), and sedative drugs aimed at sleep regulation (flurazepam).

At the moment of the study, S.T. was in the chronic phase of the illness. He demonstrated adequate cognitive skills tested by the achievement of a cut-off score of 24/30 at Mini Mental State Examination (MMSE; [44]). He had communicative-pragmatic deficits, as resulting from the administration of form A of the Assessment Battery for Communication (ABaCo; [45]) in comparison to normative data of ABaCo [46]. He attended all the training sessions. He did not suffer from anxiety disorders such as claustrophobia or panic attacks or any disorders which could be catastrophically exacerbated by confined spaces, such as MRI. Moreover, the patient met predefined exclusion criteria referring to absence of leucotomy, neurological disability, and alcohol or drug addiction. S.T. was informed about the procedure and the features of the exam and he gave his informed written consent for the rehabilitative treatment, the behavioural assessment, and the fMRI scanning. The study was approved by the Bio-Ethics Committee of the University of Turin.

### 2.2. Experimental Design

**Figure 1:**
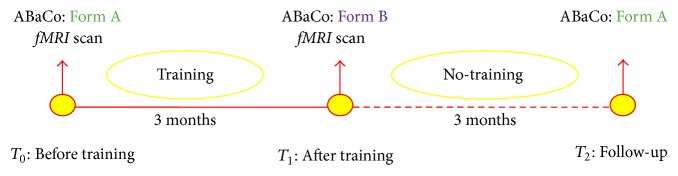
Graphical representation of the experimental design.

The study comprised a three-month training period and three experimental sessions, organized according to an ABA design as described in Figure 1.

#### 2.2.1. T0: Before Training

The patients' communicative abilities were assessed immediately before the beginning of the rehabilitation program, through the equivalent form A of ABaCo.

#### 2.2.2. T1: After Training

After completing the training program, form B of the ABaCo was administered to assess the efficacy of the training on the communicative abilities of the participants.

#### 2.2.3. T2: Follow-Up

In order to assess the stability of the patients' performance in time, form A of the ABaCo was administered again three months after the rehabilitation program.

### 2.3. The Cognitive Pragmatic Treatment

The patient took part in the CPT [47], and he underwent the fMRI scanning before and after treatment. The CPT lasted 10 weeks and it was structured in two sessions per week, each one lasting 90 minutes. Patients attended the sessions in small groups of five/six, led by a psychologist. The treatment mainly concentrated on the different expressive modalities of communication, that is, linguistic, extralinguistic, paralinguistic, social appropriateness, and conversational abilities. Moreover, other sessions were focused on aspects such as awareness, theory of mind, and planning, which have been shown to have a role to play in communicative performance [45–47]. The training provided the patients with an ecological setting where they were encouraged to exercise their communicative abilities and learn how to face typical situations of everyday interactions; this goal was realized through self-monitoring strategies and feedback provided by the therapist.

Communicative-pragmatic difficulties displayed by patients with schizophrenia are related to their difficulties in filling the gap between what is literally said and what is meant, as, for example, in indirect speech acts and figurative language (e.g., [17]). For this reason, many activities along the training were focused on the understanding of the partner's intended meaning starting from the expressed literal message. Specific attention was also given to the ability to interpret and use nonverbal and paralinguistic cues, such as facial expression and the tone of voice, in order to identify the correct speaker's communicative intention; irony, for example, is characterized by specific paralinguistic cues [48] that help a person to distinguish it from deceit. Moreover, frequently these patients have difficulty in modulating their speech according to the contextual information. This is the reason why many activities of the training were focused on the ability to decode violations of the conversational implicatures and to avoid digressions and derailments. An overview of each session is provided in Table 1. See also [34, 36] for a detailed explanation of CPT.

### 2.4. Behavioural Measures

The communicative-pragmatic abilities of the patient were measured through the administration of the equivalent forms of ABaCo [16] before and after treatment. The equivalent forms of the ABaCo have shown a good internal consistency, a good correlation between forms, and an excellent interrater agreement [15, 16]. They are comprised of four evaluation scales, that is, linguistic, extralinguistic, paralinguistic, and context, aimed at assessing the main pragmatic components of communication. Each scale is divided into two subscales, evaluating, respectively,* comprehension* and* production* abilities in each communication modality. A detailed description of the clinical tool, the procedure, and the scoring criteria is provided in [16]; see also [49].

### 2.5. fMRI Procedures

In order to investigate whether any changes in functional brain activity were detectable after CPT, we used a resting-state paradigm (rs) for imaging data. The results obtained in the pre- and posttreatment phases at the rs-fMRI analyses have been compared, in order to establish the presence of objective effects of rehabilitation at the neuronal level. Data acquisition was performed at the Koelliker Hospital in Turin. Besides the rs scanning (18 minutes), a set of anatomical MRI images were acquired (10 minutes). The patient was instructed to lie on the scanner-bed and simply keep his eyes closed, thinking of nothing in particular, and not to fall asleep.

Data acquisition was performed on a 1.5-T Philips Intera with a Sense high-field high-resolution head coil (MRIDC) optimized for functional imaging. Functional T2-weighted images were acquired using echo planar imaging (EPI) sequences, with a repetition time (TR) of 3000 ms, an echo time (TE) of 60 ms, and a 90° flip angle. The acquisition matrix was 64 × 64; the field of view (FoV) was 256 mm. For each paradigm, a total of 100 volumes were acquired. Each volume consisted of 25 axial slices, parallel to the anterior-posterior (AC–PC) commissure line and covering the whole brain; the slice thickness was 4 mm with a 0.5 mm gap. At the beginning of functional scanning, two scans were added, and their data was discarded, in order to reach a steady-state magnetization before acquisition of the actual experimental data. In the same session, a set of three-dimensional high-resolution T1-weighted structural images was acquired for each participant. This data set was acquired using a Fast Field Echo (FFE) sequence, with a repetition time (TR) of 25 ms, the shortest echo time (TE), and a 30° flip angle. The acquisition matrix was 256 × 256; the field of view (FoV) was 256 mm. The set consisted of 160 sagittal contiguous images covering the whole brain. The in-plane resolution was 1 × 1 mm and slice thickness was 1 mm (1 × 1 × 1 mm voxels).

The functional data of each subject underwent the following preprocessing steps: (1) mean intensity adjustment to prevent global signal variability; (2) slice scan time correction, using a sinc interpolation algorithm; (3) 3D motion correction: all of the volumes were aligned spatially to the first volume by rigid body transformations, using a trilinear interpolation algorithm. The subject's slice-based functional scans were coregistered to their 3D high-resolution structural scan, and the 3D structural data set was transformed into Talairach space [50]. Using the anatomical-functional coregistration matrix and the determined Talairach reference points, we transformed the functional time course into Talairach space and created the volume time course. Afterwards, the temporal series of each voxel have been filtered by a bandpass filtering (0.01 < *f* < 0.08 Hz) in order to remove both the very low frequencies and the noise due to high frequencies (respiratory and cardiac frequencies). Then the filtered time series have been transformed into a frequency domain with the Fourier transformation; this process allows decomposing a signal made of more frequencies and identifying the spectrum of the signal. The power spectrum represents the energy of the signal at different frequencies. We then calculated the Amplitude of Low Frequency Fluctuation (ALFF) index of the resting-state fMRI signal that is based on the amplitude of the low frequency fluctuations of the rs-fMRI signal and it is interpreted as reflecting the intensity of the spontaneous regional activity of the brain. The ALFF index was obtained calculating the square root of the power spectrum between 0.01 and 0.08 Hz, and it represents the average amplitude of the signal in a single voxel. In order to examine the difference between the neuronal activation before treatment and that after treatment, the functional map obtained before treatment was subtracted from that obtained after treatment, using a statistical threshold of *p* < 0.05, corrected for multiple comparisons using false discovery rate correction [51].

The script used for the analyses produces specific output able to show Brodmann areas and the cerebral gyri and sulci implicated in the functional activity changes.

## 3. Results

### 3.1. Clinical and Behavioural Outcome

The scores obtained by S.T. at the equivalent forms of ABaCo, before and after training, are displayed in Table 2, in order to get an overview of the improvement of S.T. after the rehabilitative program. The scores obtained at T0 (before training), T1 (after training), and T2 (follow-up) are compared with the means of the scores obtained at the subscales of ABaCo by the normative group with the same age and education level as S.T. [15]. As shown in the table, S.T. improved his communicative performance in all the scales of ABaCo, reaching in many cases the normative performance for his age and education level.

### 3.2. fMRI Outcome

**Figure 2:**
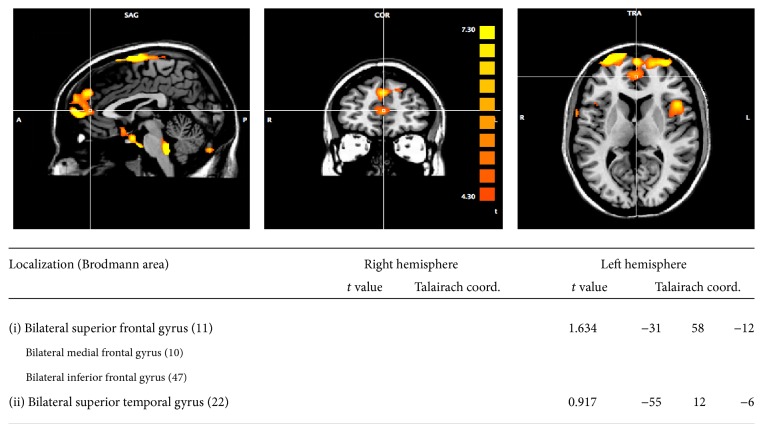
Patient R.M., post- minus pretreatment changes computed as the ALFF index. Talairach coordinates of local maxima of structures showing significant activity (*p* < 0.05, corrected for multiple comparisons). The amount of increase in the ALFF between sessions for the two clusters was 45% and 29%, respectively.

Posttraining stronger activation emerged within the superior, inferior, and medial frontal gyri, as well as the superior temporal gyri. See Figure 2.

## 4. Discussion and Conclusion

The aim of the present study was to evaluate the effect of a rehabilitative training, the CPT, focused on improving communicative-pragmatic ability and on the cerebral functional changes of a single case patient (S.T.) with schizophrenia.

As to what concerns behavioural data, the scores obtained by S.T. at the equivalent forms of ABaCo [16] before and after training were compared with the normative values of the battery for patient's age and education level [15]. S.T.'s performance was initially under the range of normative values in a number of ABaCo subscales, that is, linguistic and extralinguistic production subscales and paralinguistic and context comprehension subscales. After the CPT, S.T. improved his performance in all the subscales of ABaCo, reaching in the majority of the cases the normative level. The only exception was represented by the paralinguistic comprehension subscale, in which S.T. improved the pretraining performance, even though the obtained score was still under the normative range. A three months' follow-up testified that S.T.'s improvement persisted during this time.

Posttraining stronger activation emerged in the superior, inferior, and medial frontal gyri, as well as in the superior temporal gyri. In a study by Kircher et al. [52], the inferior frontal gyrus and the right middle temporal gyrus have been found to be involved in schizophrenic patients' impairment in understanding the figurative aspects of language, especially metaphors. Analogous results have been found by Rapp et al. [53], Eviatar and Just [54], and Shibata et al. [55, 56] who showed the involvement of the left inferior frontal cortex (BA 45,47) and of the left medial frontal cortex (BA 10) in processing metaphoric sentences. [52] suggested that the particular activation of these areas might reflect semantic-inferencing processes and that dysfunctions at this level might explain deficits in metaphor comprehension in clinical populations such as patients with schizophrenia. Moreover, the superior frontal gyrus is partially responsible for the control of executive functions and has been demonstrated to show abnormalities in schizophrenic individuals [57]. Moreover, Wang et al. [58] found the involvement of the medial frontal areas (BA 10) in the processing of ironic statements, suggesting that this recruitment could be related to the integration of the meaning conveyed by facial expressions and tone of the voice with the comprehension of the speaker's intention. Regarding discourse organization, [59] found activation in middle and superior frontal regions of the right hemisphere in discourse processing and [60] highlighted that the medial frontal regions have a role in the coherence processes in language comprehension and in establishing pragmatic connection between presented sentences. The medial frontal cortex has been found, also, to be involved in pragmatic comprehension tasks related to plausibility judgment [61] and reasoning [62]. Moreover, it seems to have a role in the evaluation of the emotional significance of the external stimuli and in mentalization processes [63], which are often impaired in schizophrenia and which have been trained during the rehabilitative sessions. Finally, regarding the modification in the superior temporal gyrus, Kuperberg et al. [64] found an involvement of this area in the elaboration of sentences which violated semantic restrictions. Moreover, Wildgruber et al. [65] indicated a broad pattern of activation related to the evaluation of linguistic and emotional aspects of speech intonation, including the prefrontal and the superior temporal cortex, that could be interpreted as related to R.M.'s training on the paralinguistic aspects of communication. It is interesting to note that our results are in line with those of a meta-analysis [66] on fMRI studies focused on nonliteral language, in terms of metaphors, proverbs, idioms, irony, and sarcasm. The authors identified a common network for nonliteral language, including left and right inferior frontal gyrus and left middle and superior temporal gyrus with contributions from medial prefrontal and superior frontal regions.

Globally considered, the detected brain modifications seem to support the behavioural improvements the patient obtained on ABaCo scales. Indeed, particular emphasis during the training has been given to the ability to go beyond the literal message of an utterance and to correctly understand the speaker's meaning. However, since this is a single case study, the results should be interpreted with caution and further research on convenience samples is necessary to confirm the findings and extend them to present as well as other clinical populations.

## Figures and Tables

**Table 1:** Structure of the Cognitive Pragmatic Treatment: topics, material, and procedures belonging to each session.

Session (s)	Topic	Material and procedure
n. 1	Awareness of one's own difficulties	Construction of the clinical setting and introduction of aims and tools of the CPT. Video-recording of the self-presentation of each patient (own communicative difficulties and expectations).

n. 2	General communicative ability: an overview	Videos and role playing tasks focused on the overall pragmatic effectiveness expressed through all the modalities constituting the communicative competence.

n. 3 n. 4	Linguistic ability	Videos and role playing tasks based on the linguistic expressive modality.

n. 5 n. 6	Extralinguistic ability	Videos and role playing tasks based on the gestural expressive modality.

n. 7 n. 8 n. 9	Paralinguistic ability	Videos, role playing tasks, and interactive activities specifically focused on facial expression recognition, rhythm, and tone of the voice.

n. 10 n. 11	Social appropriateness ability	Videos and role playing tasks focused on social appropriateness and communicative adequacy in different contexts.

n. 12 n. 13	Conversational ability	Videos, role playing tasks, and tangram exercises focused on the use of conversational rules (i.e., turn-taking and management of the topic).

n. 14	Management of conversation at the phone	Audio-taped telephone conversation and role playing tasks specifically focused on telephone conversational rules (i.e., with no possibility of taking advantage of the paralinguistic and gestural elements which usually connote communicative interactions).

n. 15	Planning ability	Individual and group activities focused on subgoals tasks (e.g., planning household chores).

n. 16 n. 17	Theory of mind	Videos and role playing tasks focused on the ability to formulate metarepresentations with respect to one's own and others' mental states.

n. 18	Narrative ability	Description tasks and speech elicitation tasks focused on the ability to tell a story or describe a situation in a proper and clear way.

n. 19	General communicative ability: a summing-up	Videos and role playing tasks focused on the overall pragmatic effectiveness expressed through all the modalities constituting communicative competence.

n. 20	Posttraining awareness	Conclusions and feedback about the results gained by every participant when compared to the initial video-recorded performance.

**Table 2:** Scores obtained by S.T. at the subscales of ABaCo at T0, before training, T1, after training, T2, follow-up, and normative values.

ABaCo subscales	Mean (DS) normative sample^a^	T0, before training	T1, after training	T2, follow-up
*Comprehension*				
Linguistic	.90 (.09)	1	1	1
Extralinguistic	.81 (.13)	.88	1	1
Paralinguistic	.89 (.09)	.67	.83	1
Context	.87 (.12)	.50	1	1
*Production*				
Linguistic	.90 (.09)	.81	1	1
Extralinguistic	.86 (.11)	.50	1	1
Paralinguistic	.98 (.04)	1	1	1
Context	.94 (.11)	1	1	1

*Note.* ^a^Means and standard deviation of the scores at ABaCo obtained by a group of healthy individuals with the same characteristics in terms of age and education level as S.T., that is, normative group ranging in age between 35 and 54 years, 13 years of education (see [15]).
